# Supplementary material for: Saccharomyces cerevisiae malate dehydrogenase Mdh1p lacking mitochondrial targeting signal can be re-localized to peroxisomes
Source: Biol Open. 2025 Sep 25;14(9):bio062199. doi: 10.1242/bio.062199 (PMC12505276; doi:10.1242/bio.062199)
Supplement: Supplementary information [file biolopen-14-062199-s1.pdf]

Table S1. List of primers used in this study

Primer	Sequence	Description	Used with	Product size
**Primers for amplifying *MDH1* gene (stop codon excluded) (for cloning into pBS35)**<br>**Template DNA: gDNA** isolated from **yeast BY4741**<br>**Resulting plasmid: pBS35-MDH1**				
CN0132	5'- GGGGTCGACATGTTGTCAAGAGTAGCTAAAC -3'	Forward primer with ***SalI*** site (underlined)	CN0133	1,020 bp
CN0133	5'- TTTCCCGGGTTTACTAGCAACAAAGTTGACAC -3'	Reverse primer with ***SmaI*** site (underlined)	CN0132	
**DNA sequencing primers (for checking the nucleotide sequence of *MDH1* after cloning into pBS35)**				
SP6	5'- ATTTAGGTGACACTATAG -3'	Universal primer (Macrogen)		
CN0138	5'- ATCCCATTGATTTCGCAAAC -3'	Forward primer; located at **nt601-620** of ***MDH1*** coding sequence		
**Primers for preparing DNA cassette** [carrying **50-bp upstream** sequence of ***MDH1* start codon** + **ATG** + ***MDH1*** coding sequence **without MTS** sequence (**nt4-51Δ**) (no stop codon) + linker sequence + **mCherry** coding sequence + hygromycin resistance gene + **50-bp downstream** sequence of ***MDH1* stop codon**] **(used for transforming yeast BY4741** or **yeast *ALD4(noMTS)::GFP; kanR*)**<br>**Template DNA: pBS35-MDH1**<br>**Resulting yeast strains:**<br>(1) ***MDH1(noMTS)::mCherry; hygR*** (BY4741 as a background) and<br>(2) ***MDH1(noMTS)::mCherry; hygR*** [*ALD4(noMTS)::GFP; kanR* as a background]				
CN0135	5'- TACGGAAAGGAAGAAAAAAAACAAAAGGAAAAGGAAGGATACCATATACAATGTATAAAGTGACTGTTTTGGGTG -3'	Forward primer containing **50-nt upstream** sequence of ***MDH1*** start codon + **ATG** (underlined) + **nt52-73** of ***MDH1*** coding sequence	CN0123	3,468 bp
CN0123	5'- TTTTTTTTTTTTTTTCCCTATTTTTCACTCTATTTCTGATCTTGAACAATATCGATGAATTCGAGCTCG -3'	Reverse primer containing **50-nt downstream** sequence of ***MDH1* stop codon** (reverse-complement) + sequence located after **hphMX6** of **pBS35** (reverse-complement; underlined)	CN0135	
**Primers for preparing PCR samples for yeast strain verification by DNA sequencing** (Macrogen) **to validate the successful deletion of MTS from *MDH1* gene and introduction of mCherry downstream of *MDH1***<br>**Template DNA: gDNA** isolated from **yeast BY4741** or **yeast *ALD4(noMTS)::GFP; kanR*** transformed with the **DNA cassette** [carrying **50-bp upstream** sequence of ***MDH1* start codon** + **ATG** + ***MDH1*** coding sequence				

Biology Open • Supplementary information

Primer	Sequence	Description	Used with	Product size
**without MTS** sequence (**nt4-51Δ**) (no stop codon) + linker sequence + **mCherry** coding sequence + hygromycin resistance gene + **50-bp downstream** sequence of ***MDH1* stop codon**]				
CN0128	5’- AAGTTGCCATAATACAATGGCGG -3’	Forward primer; located at **200-nt upstream** of ***MDH1* start codon**	CN0141	2,002 bp (**if still having MTS**) or 1,954 bp (**if no MTS**)
CN0141	5’- GGTATTCTGGGCCTCCATGTC -3’	Reverse primer; located at **nt1-21** of **hphMX6** sequence (within **pBS35**; reverse-complement)	CN0128	
**DNA sequencing primers (for checking the successful deletion of MTS from *MDH1* gene and introduction of mCherry coding sequence downstream of *MDH1*)**				
CN0128	5’- AAGTTGCCATAATACAATGGCGG -3’	Forward primer; located at 200-nt upstream of ***MDH1*** start codon		
CN0141	5’- GGTATTCTGGGCCTCCATGTC -3’	Reverse primer; located at nt1-21 of hphMX6 sequence (within **pBS35**; reverse-complement)		
**Primers for preparing DNA cassette** [carrying **50-bp upstream** sequence of ***MDH2* stop codon** + linker sequence + **GFP** coding sequence + kanamycin resistance gene + **50-bp downstream** sequence of ***MDH2* stop codon**] **(used for transforming yeast *MDH1(noMTS)::mCherry; hygR*)**<br>**Template DNA: pFA6a-GFP(S65T)-kanMX6**<br>**Resulting yeast strain: *MDH2::GFP; kanR*** [*MDH1(noMTS)::mCherry; hygR* as a background]				
CN0142	5’- AAAATATCGATAAGGGCTTGGAATTCGTTGCATCGAGATCTGCATCATCTGGTCGACGGATCCCCGGG -3’	Forward primer containing **50-nt upstream** sequence of ***MDH2* stop codon** + sequence located in front of **GFP** coding sequence of **pFA6a-GFP(S65T)-kanMX6** (underlined)	CN0143	2,536 bp
CN0143	5’- ACGGGAATATTATCAATTTGCTGCATTCTTATGCTTCGGTCCGATGCTCAATCGATGAATTCGAGCTCG -3’	Reverse primer containing **50-nt downstream** sequence of ***MDH2* stop codon** (reverse-complement) + sequence located after **kanMX6** of **pFA6a-GFP(S65T)-kanMX6** (reverse-complement; underlined)	CN0142	
**Primers for verifying the successful insertion of GFP coding sequence downstream of *MDH2* gene**<br>**Template DNA: gDNA** isolated from **yeast *MDH1(noMTS)::mCherry; hygR*** transformed with the **DNA cassette** [carrying **50-bp upstream** sequence of ***MDH2* stop codon** + linker sequence + **GFP** coding sequence + kanamycin resistance gene + **50-bp downstream** sequence of ***MDH2* stop codon**]				
CN0146	5’- TACACGCGCGTGCGTAGATATATAAAG -3’	Forward; located 200 nt upstream of ***MDH2*** start codon	JW1623	2,256 bp
JW1623	5’- GCGACCTCATACTATACCTG -3'	Reverse primer; located between **GFP** and **kanMX6** of **pFA6a-GFP(S65T)-kanMX6** (**164-nt downstream** of **GFP stop codon**) (reverse-complement)	CN0146	

Biology Open • Supplementary information

Primer	Sequence	Description	Used with	Product size
**Primers for preparing DNA cassette** [carrying **50-bp upstream** sequence of ***PEX3* stop codon** + linker sequence + **GFP** coding sequence + kanamycin resistance gene + **50-bp downstream** sequence of ***PEX3* stop codon**] **(used for transforming yeast *MDH1(noMTS)::mCherry; hygR*)**<br>**Template DNA: pFA6a-GFP(S65T)-kanMX6**<br>**Resulting yeast strain: *PEX3::GFP; kanR*** [*MDH1(noMTS)::mCherry; hygR* as a background]				
CN0148	5'-CCAGCGTATACAGCAACTTTGGCGTCTCCAGCTCGTTTTCCTTCAAGCCTGGTCGACGGATCCCCGGG -3'	Forward primer containing **50-nt upstream** sequence of ***PEX3* stop codon** + sequence located in front of **GFP** coding sequence of **pFA6a-GFP(S65T)-kanMX6** (underlined)	CN0149	2,536 bp
CN0149	5'-ACGCTATATATATATATATTCTGGTGTGAGTGTCAGTACTTATTCAGAGAATCGATGAATTCGAGCTCG -3'	Reverse primer containing **50-nt downstream** sequence of ***PEX3* stop codon** (reverse-complement) + sequence located after **kanMX6** of **pFA6a-GFP(S65T)-kanMX6** (reverse-complement; underlined)	CN0148	
**Primers for verifying the successful insertion of GFP coding sequence downstream of *PEX3* gene**<br>**Template DNA: gDNA** isolated from **yeast *MDH1(noMTS)::mCherry; hygR*** transformed with the **DNA cassette** [carrying **50-bp upstream** sequence of ***PEX3* stop codon** + linker sequence + **GFP** coding sequence + kanamycin resistance gene + **50-bp downstream** sequence of ***PEX3* stop codon**]				
CN0152	5'- ATGTCGTTAACAAGTAGTAGAGTTTG -3'	Forward primer; located **200-nt upstream** of ***PEX3* stop codon**	JW1623	2,448 bp
JW1623	5'- GCGACCTCATACTATACCTG -3'	Reverse primer; located between **GFP** and **kanMX6** of **pFA6a-GFP(S65T)-kanMX6** (**164-nt downstream** of **GFP stop codon**) (reverse-complement)	CN0152	

Table S2. Assembly frequency analysis of the MTS-lacking Mdh1p (tagged with mCherry) in the yeast construct "*MDH1(noMTS)::mCherry*"

	**Clone #**	**% cells with Mdh1p(noMTS)-mCherry structures** (irrespective of their localization)		
		**Log phase**	**1-day culture (saturation)**	**5-day culture (stationary phase)**
**Exp. 1**	**1**	100.00 (256 cells*)	100.00 (251 cells*)	100.00 (285 cells*)
	**2**	100.00 (251 cells*)	100.00 (272 cells*)	100.00 (291 cells*)
**Exp. 2**	**1**	100.00 (254 cells*)	100.00 (253 cells*)	100.00 (271 cells*)
	**2**	100.00 (256 cells*)	100.00 (271 cells*)	99.62 (263 cells*)
**Exp. 3**	**1**	100.00 (261 cells*)	100.00 (265 cells*)	100.00 (261 cells*)
	**2**	100.00 (256 cells*)	100.00 (264 cells*)	100.00 (266 cells*)
**Average ± SEM**	**1**	**100.00 ± 0.00**	**100.00 ± 0.00**	**100.00 ± 0.00**
	**2**	**100.00 ± 0.00**	**100.00 ± 0.00**	**99.87 ± 0.13**

Notes:

(1) Structures of Mdh1p(noMTS)-mCherry were observed within the mitochondria of yeast samples grown to log-phase.

(2) The asterisk (*) indicates the total number of cells counted.

Biology Open • Supplementary information

Table S3. Co-localization frequency analysis of MTS-lacking Ald4p (tagged with GFP) and MTS-lacking Mdh1p (tagged with mCherry) in the yeast construct "*ALD4(noMTS)::GFP* and *MDH1(noMTS)::mCherry*"

5-day culture (stationary phase)	Clone #	% cells showing both Ald4p(noMTS)-GFP and Mdh1p(noMTS)-mCherry structures	% cells showing either Ald4p(noMTS)-GFP or Mdh1p(noMTS)-mCherry structures	% cells without any structures	Total number of cells (used for counting)
**Exp. 1**	**1**	65.26 (0% colocalization)	21.15	13.60	331
	**2**	67.09 (0% colocalization)	18.04	14.87	316
**Exp. 2**	**1**	64.81 (0% colocalization)	15.74	19.44	324
	**2**	67.63 (0% colocalization)	12.50	19.87	312
**Exp. 3**	**1**	65.70 (0% colocalization)	17.80	16.50	309
	**2**	67.77 (0% colocalization)	18.07	14.16	332
**Average ± SEM**	**1**	**65.26 ± 0.25** (0% colocalization)	**18.23 ± 1.58**	**16.51 ± 1.69**	**-**
	**2**	**67.50 ± 0.21** (0% colocalization)	**16.20 ± 1.85**	**16.30 ± 1.80**	**-**

Biology Open • Supplementary information

Table S4. Co-localization frequency analysis of MTS-lacking Mdh1p (tagged with mCherry) and Mdh2p (tagged with GFP) in the yeast construct "*MDH1(noMTS)::mCherry* and *MDH2::GFP*"

1-day culture (saturation)	Clone #	% cells showing both Mdh1p(noMTS)-mCherry and Mdh2p-GFP structures	% cells showing either Mdh1p(noMTS)-mCherry or Mdh2p-GFP structures	% cells without any structures	Total number of cells counted
**Exp. 1**	**1**	71.05 (100% colocalization)	21.05	7.89	152
	**2**	70.63 (100% colocalization)	20.00	9.38	160
**Exp. 2**	**1**	71.34 (100% colocalization)	18.29	10.37	164
	**2**	70.44 (100% colocalization)	20.75	8.81	159
**Exp. 3**	**1**	71.43 (100% colocalization)	18.83	9.74	154
	**2**	70.70 (100% colocalization)	19.75	9.55	157
**Average ± SEM**	**1**	**71.27 ± 0.11** (100% colocalization)	**19.39 ± 0.84**	**9.33 ± 0.74**	**-**
	**2**	**70.59 ± 0.08** (100% colocalization)	**20.17 ± 0.30**	**9.24 ± 0.23**	**-**

Biology Open • Supplementary information

Table S5. Co-localization frequency analysis of MTS-lacking Mdh1p (tagged with mCherry) and Pex3p (tagged with GFP) in the yeast construct "*MDH1(noMTS)::mCherry* and *PEX3::GFP*"

1-day culture (saturation)	Clone #	% cells showing both Mdh1p(noMTS)-mCherry and Pex3p-GFP signals	% cells showing either Mdh1p(noMTS)-mCherry or Pex3p-GFP signals	% cells without any structures or signals	Total number of cells counted
**Exp. 1**	**1**	72.33 (100% colocalization)	20.75	6.92	159
	**2**	71.60 (100% colocalization)	23.46	4.94	162
**Exp. 2**	**1**	72.61 (100% colocalization)	21.02	6.37	157
	**2**	71.43 (100% colocalization)	22.36	6.21	161
**Exp. 3**	**1**	72.55 (100% colocalization)	20.68	6.83	153
	**2**	71.70 (100% colocalization)	22.61	6.29	159
**Average ± SEM**	**1**	**72.50 ± 0.09** (100% colocalization)	**20.68 ± 0.22**	**6.83 ± 0.24**	**-**
	**2**	**71.58 ± 0.08** (100% colocalization)	**22.61 ± 0.44**	**5.81 ± 0.44**	**-**
